# Effects of Low Temperature, Freeze–Thaw Cycles, and Healing Conditions on Viability of Non-Ureolytic Bacteria in Biological Self-Healing Concrete

**DOI:** 10.3390/ma17235797

**Published:** 2024-11-26

**Authors:** Augusta Ivaškė, Ronaldas Jakubovskis, Renata Boris, Jaunius Urbonavičius

**Affiliations:** 1 Department of Chemistry and Bioengineering, Faculty of Fundamental Sciences, Vilnius Gediminas Technical University (VILNIUSTECH), Saulėtekio al. 11, 10223 Vilnius, Lithuania; augusta.ivaske@vilniustech.lt; 2 Institute of Building and Bridge Structures Laboratory of Innovative Building Structures, Vilnius Gediminas Technical University, 10223 Vilnius, Lithuania; ronaldas.jakubovskis@vilniustech.lt; 3 Laboratory of Composite Materials, Institute of Building Materials, Vilnius Gediminas Technical University (VILNIUSTECH), 10223 Vilnius, Lithuania

**Keywords:** biological concrete, self-healing concrete, low temperatures, freeze–thaw testing

## Abstract

The capacity of biological self-healing concrete (BSHC) to repair cracks relies on the sustained viability and metabolic function of bacteria embedded within the concrete. BSHC structures face significant risk in cold climates due to low temperatures and freeze–thaw (FT) cycles, during which freezing water can generate internal pressure that damages bacterial cells and diminishes their activity. A special feature of this study is the incorporation of bacterial spores within expanded clay aggregates, tested under varying environmental conditions. The viability of bacterial spores was measured under cold and freeze–thaw cycles by counting colony-forming units, and a specific methodology was developed to assess the efficiency of self-healing under rain-simulated conditions. It was demonstrated that bacteria embedded in concrete could endure fluctuations in low temperatures and freeze–thaw cycles, compromising approximately 50% of viable spores. Also, it was found that water immersion during concrete curing can trigger early germination, decreasing viable spore counts by nearly tenfold. Ultimately, it was demonstrated that the healing of cracks in BSHC components is influenced by the conditions under which the specimens are incubated. The results suggest that BSHC can be employed in cold climate areas, given that suitable curing conditions and adequate bacterial protection within the concrete are ensured.

## 1. Introduction

Concrete remains the most extensively utilized structural material. It provides excellent compressive strength and satisfactory workability at a relatively affordable cost. However, its tensile strength is significantly lower, about 10–15 times less than its compressive strength. Consequently, the tension areas in concrete structures with reinforcement frequently develop cracks, permitting the entry of water, oxygen, and salts. In turn, this leads to swift corrosion of reinforcement and degradation of concrete. Cracks have been recognized as the most damaging flaw in concrete structures [1].

Using self-healing concrete is an economical and sustainable method for managing damage in concrete structures. Over the past few decades, various self-healing strategies have been devised for concrete, including the micro- or macro-encapsulation of healing agents, biologically based healing methods, the incorporation of mineral admixtures, vascular systems, and shape-memory alloys [2]. Out of these techniques, the biological approach is regarded as one of the most environmentally friendly and effective methods for concrete healing [3,4,5,6]. The microencapsulation of bacteria involves enclosing bacterial spores within a capsule, which can protect the bacteria from harsh environmental conditions. Macroencapsulation is a larger-scale encapsulation, such as embedding bacteria in aggregates, which can be incorporated into a concrete matrix. Typical protective materials are expanded clay, perlite, diatomaceous earth and hydrogel [7,8,9]. These carriers protect bacteria from harsh concrete environments such as high pH and mechanical stress during concrete mixing and hardening.

Despite the use of bacteria in self-healing concrete, one more application of using microbially induced calcite precipitation (MICP) is to improve soil reinforcement. MICP is a promising method for soil reinforcement that utilizes bacteria to promote the formation of calcium carbonate, which can enhance soil stability and reduce permeability. This technique has gained attention as a sustainable and environmentally friendly approach to soil improvement. MICP operates by leveraging bacterial metabolic processes, primarily ureolytic bacteria, to precipitate calcite, which binds soil particles together, improving soil strength and stiffness. This method has shown considerable promise in controlled laboratory settings [10]. However, challenges remain in translating them to field applications due to variability in soil composition, bacterial survival in diverse environmental conditions, and potential regulatory concerns related to introducing bacteria or enzymes into the soil.

Incorporating specific microorganisms into concrete composites has been shown to enhance the material’s self-healing capabilities and durability. Bacteria such as *Bacillus* species can precipitate calcium carbonate in microcracks, reducing permeability and extending the lifespan of concrete structures. This approach has been increasingly combined with advanced materials to further improve performance under environmental stresses like sulfate exposure and freeze–thaw cycles. Recent studies explore the role of fly ash and nano-silica in cementitious composites. These materials not only enhance the fracture mechanics parameters of concrete but also improve its homogeneity and resistance to cracking. Fly ash acts as a pozzolan, reacting with calcium hydroxide to form additional calcium silicate hydrate (C-S-H), thus enhancing the microstructure.

Nano-silica, with its high specific surface area, can further densify the matrix and reduce porosity, resulting in higher strength and fracture toughness [11]. Thermoplastic microcapsules have been investigated as additives in concrete to improve its self-healing capabilities, particularly in high-sulfate environments. It was shown that microcapsules provide an effective mechanism for sealing cracks when they break and release healing agents in response to stress. The use of these microcapsules, combined with microbial healing agents, could lead to enhanced performance by tackling both immediate crack healing (from microcapsules) and long-term durability improvements (from microbial action) [12]. The integration of microbial self-healing mechanisms with materials such as fly ash, nano-silica, and microcapsules in cementitious concrete can create synergistic effects. These combinations may optimize the material’s performance by providing multi-faceted protection and repair mechanisms.

The process of calcite deposition facilitated by microorganisms forms the foundation of concrete capable of biologically self-healing. Nonetheless, the bacteria must endure the challenging conditions found in concrete. Consequently, the bacteria most frequently utilized for bio-concrete production are alkali tolerant and capable of forming spores [13]. Three primary types of bacteria can induce calcium carbonate precipitation: ureolytic, non-ureolytic, and denitrifying bacteria. Nitrate-reducing bacteria that function in environments with limited oxygen have been employed in biological concrete to generate calcium carbonate. Ureolytic bacteria produce urease, the enzyme responsible for precipitating calcium carbonate. A drawback of this method is the emission of ammonia into the surroundings, which heightens the risk of reinforcement corrosion [14,15]. An alternative nutrient source has been discovered to resolve the issues arising from urea hydrolysis. Non-ureolytic bacteria can induce calcium carbonate precipitation without emitting ammonium ions. The Bacillus genus is also recognized for its capability to precipitate calcium carbonate [14,16].

Biological self-healing concrete (BSHC) utilizes bacteria that precipitate calcium carbonate. As cracks form, oxygen and water infiltrate the concrete, triggering the metabolic activities of the bacteria [17]. Thus, a critical component in the creation of BSHC involves ensuring the bacteria embedded within the concrete remain viable and metabolically active over time [18]. Concrete presents a challenging environment for microorganisms, with elevated pH levels and the mechanical stress exerted on cells during the curing phase [10,14,19]. Furthermore, the cement used in making concrete might have harmful metal oxides that curb bacterial proliferation [20]. Bacterial spores need supplementary protection, like particular carriers or capsules, to survive within the concrete matrix [21]. Our previous investigations demonstrated that using magnesium oxide or styrene-acrylate emulsion as coating materials could improve bacterial survival rates by almost ten times [22]. Even though capsules and coating materials may provide adequate long-term survival for bacteria in concrete, BSHC structures inevitably face the impact of temperature variations and freeze–thaw (FT) cycles in colder climates. Ice formation can create added internal pressure and stress conditions, potentially harming the bacterial cells and reducing viability [23,24,25]. To fully realize BSHC advantages, the bacteria embedded in the concrete need to withstand both the challenging conditions present in concrete and the repeated temperature variations along with freeze–thaw cycles.

Studies have primarily focused on how sub-zero temperatures and freeze–thaw cycles affect the viability of soil bacteria [25,26,27,28]. Microorganisms in the upper layer of soil are constantly subjected to fluctuations in temperature and FT cycles. It was reported that lowering temperatures up to −45 °C in normal air conditions has a minor effect on bacterial viability [27]. However, when bacteria were subjected to FT cycles, the number of viable bacteria significantly decreased. It was reported that the viability of model bacteria *B. subtilis* decreased from 30% to 100% after 10 days of FT cycling [25]. The number of viable bacterial spores mostly decreases after the first FT cycle and remains stable after 5 and 10 cycles. The initial reduction in viability was attributed to the loss of cell membrane integrity, as bacterial viability well correlated with the damage of cells.

A mix of bacteria containing 30 soil bacteria species was tested for FT cycling many years ago [26]. A reduction of viability of about 50% was obtained when freezing temperatures were varying from −9 °C to −27 °C. This reduction was almost constant from the second to the 10th FT cycle, similar to what was observed by [25]. The viability of Arctic tundra soil bacterial species was also studied previously [27]. It was concluded that the bacterial community was only a little affected by temperature fluctuation and FT cycles. The ability of bacteria to survive under extreme temperature fluctuations and FT cycles may be attributed to specific bacterial survival strategies such as biofilm formation or motility [25]. As a result, it may explain the high percentage of viable bacterial cells even after 10 FT cycles.

Fewer research initiatives have focused on examining the impact of cold environments and repeated freezing and thawing processes on bacteria incorporated within concrete. Some studies have indicated that certain bacterial species can actively form CaCO_3_ in concrete close to freezing temperatures, specifically around 4 to 7 °C [28,29]. Previous work [30] identified the best amount of self-healing agents for immobilized microbial spores for concrete frost resistance. When there is no self-healing agent, the free water in the capillaries freezes and expands, which may cause tensile stress and increase both the connected porosity and the amount of microcracks in the concrete. The impact of sub-zero temperatures on bacterial viability was studied [31]. Research demonstrated that bacteria embedded in expanded clay (EC) could withstand temperatures down to −20 °C for up to 60 days. However, there are limited data on bacterial survival under repeated temperature changes and freeze–thaw (FT) cycles. The current study examined the survival of bacteria in BSHC samples when subjected to low temperatures as well as FT cycles. The effect of concrete curing conditions on the quantity of viable bacterial cells was also assessed. Ultimately, it was demonstrated that the healing process relies not only on the number of viable cells in BSHC but also on the environmental healing circumstances. The main elements of BSHC are microorganisms immobilized in expanded clay. They allow for cracks to fill over a period of time, improve mechanical properties, and reduce repair costs for concrete construction. Also, it is important to promote the industrial upgrading of the new building materials. 

Studies on microorganisms embedded in concrete are still needed to improve the properties of biological self-healing concrete. This work is the first step in investigating the use of different bacterial spores for their immobilization into expanded clay aggregates under different environmental conditions. Research in these areas helps to ensure that BSHC is applicable not only in mild climates but also in regions with harsh climates, thus extending the lifetime of concrete structures and reducing their maintenance costs. The novelty of this work lies in the exploration of bacterial survival and crack healing ability in biological self-healing concrete (BSHC) under harsh environmental conditions, particularly low temperatures and freeze–thaw (FT) cycles. This work is significant because it demonstrates that BSHC can be effectively used in cold climates, provided that adequate protection and proper curing conditions are implemented, thus expanding the potential application of self-healing concrete in colder regions where freeze–thaw damage is a concern.

## 2. Materials and Methods

### 2.1. Healing Agent Preparation

#### 2.1.1. Bacterial Growth and Spore Preparation

Two strains of bacteria, *Bacillus pseudofirmus* (DSM 8715) and *Bacillus cohnii* (DSM 6307) were acquired from the German Collection of Microorganisms and Cell Cultures (DSMZ). These bacterial strains were chosen for their excellent sporulation yields and capability to thrive within the concrete matrix [32]. As per the manufacturer’s instructions, the bacteria were regularly grown in an alkaline nutrient medium. This medium comprised 5 g/L of peptone, 3 g/L of meat extract, 0.42 g/L of NaHCO_3_, and 0.53 g/L of Na_2_CO_3_. The bacteria were then incubated overnight at a temperature of 30 °C with continuous shaking at 150 rpm.

Spores were prepared in a sporulation medium containing 3.5 g/L sucrose, 4 g/L yeast extract, 0.02 g/L KH_2_PO_4_, 0.166 g/L CaCl_2_, 0.476 g/L KCl, 0.2 g/L MgSO_4_, 0.2 g/L MnSO_4_, 4.2 g/L Na_2_CO_3_, and 5.3 g/L NaHCO_3_. A spore suspension was obtained by inoculating the overnight bacterial culture into the sporulation medium. The bacterial cultures were grown under aerobic conditions at 30 °C with agitation at 150 rpm until they reached a density of 10^9^ cells/mlL. Sporulation was examined using light microscopy after the application of the Shaeffer–Fulton staining method [16]. After 4 days of growth, spore-rich cultures were harvested by centrifugation. The spore suspension underwent two washing steps with a sterile 10 mM Tris-HCl buffer (pH 9). To remove vegetative cells, the bacteria culture was subjected to heating at 80 °C for 30 minutes and washed twice. The spore suspension was then diluted in series using the washing buffer. Suitable dilutions were plated onto alkaline nutrient agar and incubated overnight at 30 °C. Colony counts were conducted after 24 h.

#### 2.1.2. Bacteria Immobilization into Expanded Clay

Expanded clay (EC, Liapor 4–8 mm) was impregnated by a vacuum pressure of 0.1 MPa. The impregnation solution was composed of 80 g/L of calcium lactate, 1 g/L yeast extract, and 1 × 10^8^ CFU/mL of bacterial spores. EC particles were dried at room temperature for 72 h until the constant mass. To determine the number of viable bacteria in expanded clay, 1 g of expanded clay particles was ground to a fine powder suspended in 10 mM Tris-HCl buffer (pH 9) and homogenized by vortexing. Then, serial dilutions were made. The aliquots of suitable dilutions were spread onto Petri plates with a solid alkaline nutrient medium and incubated at 30 °C overnight. Grown colonies were counted, and the number of viable bacteria cells was calculated. On average, 4.2 × 10^5^ CFU/mL of *B. pseudofirmus* and 4.45 × 10^5^ CFU/mL of *Bacillus cohnii* were immobilized in one gram of dry EC.

To provide protection to the bacterial spores, the EC particles were coated with a styrene-acrylate emulsion (Weberfloor 4716, Saint-Gobain, Paris, France). The expanded clay particles were immersed in a uniquely formulated protective solution to ensure even distribution of the coating. Afterward, the EC particles with the coating were allowed to air-dry at room temperature for 48 h.

### 2.2. Preparation of BSHC Samples

#### 2.2.1. Preparation of BSCH Samples

**Figure 1:**
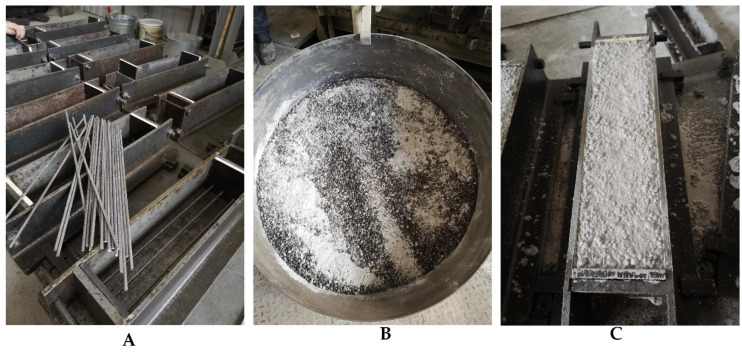
Preparation of structural biological concrete specimens: (**A**) placement of reinforcement; (**B**) dry components in a rotating pan mixer; (**C**) specimens after casting.

**Figure 2:**
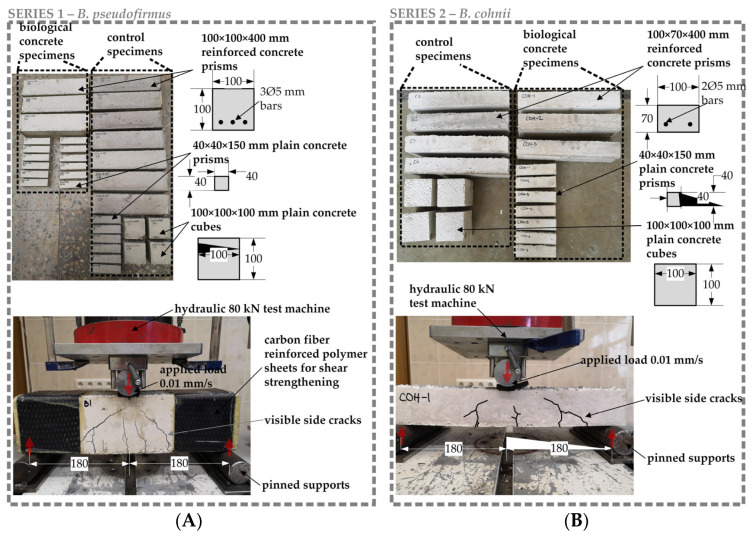
Preparation of BSHC samples: (**A**) specimens of Test Series 1; (**B**) specimens of Test Series 2.

Biological concrete specimens were produced using ordinary white Portland cement (Aalborg White^®^, Aalborg Øst, Denmark). This cement was selected because it contains reduced levels of toxic metallic oxides, which serve as bacterial inhibitors [20]. Natural gradation sand (0/4 mm) and expanded clay (Liapor 4–8 mm, Liapor GmbH, Hallendorf, Germany) were used as fine and coarse aggregates, respectively. The batch of concrete detailed in Table 1 was utilized for each series of tests. Initially, the dry ingredients were placed in a rotating pan mixer (Zyklos ZZ 75 HE, Pemat, Freisbach, Germany) and combined for 1 min (Figure 1B). Subsequently, water was added, and the mixing continued for an extra two minutes. Prepared concrete specimens were left to cure for 28 days (Figure 1C). BSHC samples were produced in two sets. In the first series, the *B. pseudofirmus* bacteria strain was used (Figure 2A). *B. cohnii* (Figure 2B) was used in the second series.

As shown in Figure 2, the reinforced or plain BSHC specimens were cast. Reinforced BSHC prisms were utilized to assess the crack healing process, while plain concrete samples were used for testing the viability of bacteria (Section 2.4). Control samples of identical dimensions were also created for laboratory testing (Figure 2). For these control samples, the concrete mix outlined in Table 1 was used, but without the inclusion of bacterial spores.

A larger number of samples in Test Series 1 were used to calibrate the load and crack opening. From Test Series 1, we found that shear reinforcement is required to achieve the necessary crack widths (>0.1 mm). Several control specimens were loaded until full failure. Consequently, we reduced the height of structural specimens in the second test series. This allowed us to obtain the necessary crack widths in all specimens without shear failure.

#### 2.2.2. Crack Opening in BSCH Specimens

**Figure 3:**
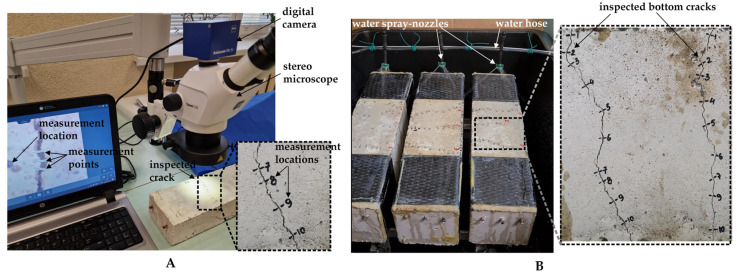
Evaluation of healing: (**A**) crack measurement scheme; (**B**) healing conditions.

Following a 28-day period of water immersion for curing, cracks in the reinforced specimens were generated through a three-point loading method. To prevent shear failure, reinforced concrete specimens of Test Series 1 were additionally strengthened with carbon fiber sheets (Figure 2A). For Test Series 2 (Figure 2B), the height of the prisms was reduced, and the additional shear strengthening was unnecessary. On average, four visible cracks formed at the bottom of the prisms. Cracks exceeding 50 µm in width were chosen for additional examination. The initial widths of these cracks were determined using a Zeiss Stemi 305 stereo microscope fitted with an AxioCam ERc 5s 5.0-megapixel digital camera. For every crack, 8 to 10 measuring spots were identified, ensuring parallel cracks, absent aggregates, and other anomalies were avoided. At each location, the width of the crack was gauged at three distinct points (Figure 3A).

Next, reinforced concrete specimens were moved to the specifically designed rain-simulating basins for a long-term incubation (healing) period (Figure 3B). The specimens were automatically sprayed with water for 30 min twice daily. After each dry cycle, water gradually drained and evaporated from the bottom side of the specimens. For the remainder of the time, the specimens were maintained in an environment with relative humidity (RH) ranging from 40% to 50%. The widths of the cracks were assessed after 28 and 98 days of the healing phase.

### 2.3. Freeze–Thaw Testing

**Figure 4:**
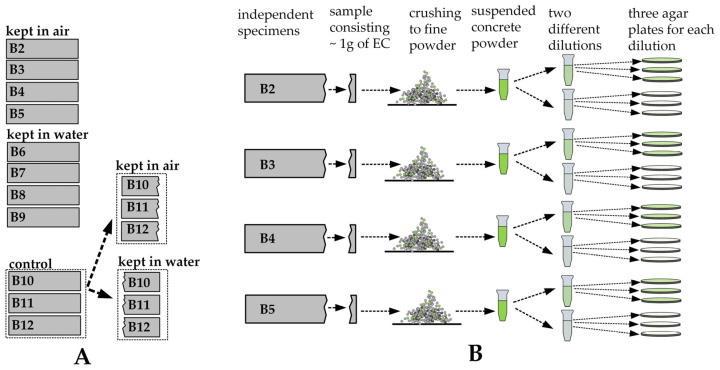
Bacterial viability tests: (**A**) specimens used for FT and temperature fluctuation tests; (**B**) schematic illustration of viability tests.

Four 40 mm × 40 mm × 150 mm BSHC specimens were placed in water and exposed to the repeated freeze–thaw cycles. One freeze–thaw cycle involved spending 2–4 days in a freezer at −20 °C, followed by 1 day of thawing at room temperature (20 °C). Twenty freeze–thaw cycles were completed in 78 days. Additionally, four BSHC specimens were exposed to fluctuation ranging from −20 °C to 20 °C. The procedure was the same as the FT tests, but the specimens were stored in air. Three specimens were maintained at a constant room temperature, either in air or in water, serving as a control (Figure 4A).

### 2.4. Bacterial Viability Testing

The viability of bacterial spores within the concrete matrix was assessed following fluctuations in temperature lasting for 1, 3, 5, 10, 15, and 20 cycles. For each viability test, a section of concrete was extracted from the 40 mm × 40 mm × 150 mm specimen and ground into powder. Subsequently, 6.53 g of this powder, which contained about 1 g of bacteria-embedded EC, was measured. A viability test included samples from four BSHC samples, as illustrated in Figure 4B. The powdered concrete was mixed in a sterile 10 mM Tris-HCl buffer (pH 9) and homogenized by vortexing. The samples were then serially diluted within the same buffer. Serial dilution was utilized (10–1000-fold) to achieve roughly 20–200 colony-forming units (CFUs) on each agar plate. Aliquots of the appropriate dilutions were seeded on an agarized nutrient medium and incubated at 30 °C for 16–18 h. The resulting colonies were counted after 24 h. The number of colonies grown on the agar plate was multiplied by the dilution factor to determine the CFU of the original sample.

### 2.5. Variation of Concrete Curing Conditions

Six cylindrical concrete specimens, each measuring 59 mm in diameter and 15 mm in height, were cast using the same concrete mix as described in Section 2.2 with *B. pseudofirmus* bacterial strain. Concrete samples were prepared in individual plastic cups, sealed, and allowed to cure at room temperature for one day. Then, three specimens were immersed in water, whereas another three were kept in air (RH ~ 50%). All specimens were kept at the same room temperature (20 °C). Bacterial viability was determined after 1, 7, 14, 21, and 28 days following the procedure outlined in Section 2.4.

## 3. Results and Discussion

### 3.1. Survival of Bacteria in Cold Environments and During Freezing and Thawing Processes

**Figure 5:**
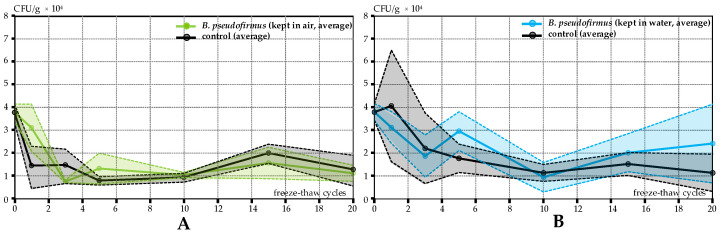
Survival of bacteria in concrete from Test Series 1: (**A**) changes in CFU due to low-temperature cycles; (**B**) changes in CFU due to FT cycles. Continuous lines represent the average values of the calculated CFU/g, whereas the shaded areas show the 95% confidence intervals.

This study further investigated how environmental conditions impact bacterial viability [33]. Figure 5A shows the change in colony-forming units (CFU) in response to cycles of low temperatures. Initially, the number of CFU in specimens subjected to low temperatures reduced by approximately 50 to 60% after multiple cycles. Subsequently, the number of viable cells stabilized and remained nearly constant until the 20th cycle (around 1.5 × 10^4^). These results align well with previous research that examined soil bacteria viability at low temperatures [26,27]. Overall, the CFU count was comparable in both control and low-temperature affected specimens. This indicates that EC-embedded bacteria within concrete might endure the repetitive impact of sub-zero temperatures. As a result, the BSHC structure could retain its healing capability even in cold climates. However, the expansion of freezing water in saturated concrete could create additional internal pressure, causing damage to the bacterial cells and reducing their viability [20].

The findings regarding bacterial survival in specimens exposed to freeze–thaw cycles are depicted in Figure 5B. In a like manner, for samples impacted by cold conditions, the starting CFU count decreased by about 50% following several initial FT cycles and stayed constant up to the 20th cycle. This trend was observed in both the water-maintained samples (serving as a positive control) and the specimens subjected to FT cycles, suggesting that the decline in viable spores was more due to concrete aging than the freezing and thawing process. Comparable outcomes were similarly noted for the *B. cohnii* bacterial strain.

The bacterial viability in specimens subjected to low temperatures and freeze–thaw conditions showed widely varied results. In certain instances, the counted CFU numbers even rose over time. A similar pattern was noted in [26], where the increase in viable bacterial spores was associated with slow bacterial growth throughout freezing cycles. In this situation, though, it is more plausible that the fluctuations in CFU are due to the irregular distribution of bacteria-immobilized expanded clay particles within the concrete. The nature of viability testing and sample preparation (see Section 2.4) makes the data dependent on the amount of healing agent (bacteria-immobilized EC particles) that is collected in concrete samples. To improve the consistency and reliability of colony-forming units’ measurement in the concrete samples containing immobilized expanded clay particles, future research could focus on taking multiple subsamples from different locations within each specimen. This would help account for local variations in the distribution of EC particles and bacteria, leading to a more representative overall CFU count. Nevertheless, there were no significant differences between the calculated CFU in any treatment through the course of the experiments. Specimens exposed to low temperatures, freeze–thaw cycles, and control conditions demonstrated similar CFU variation over time. These results suggest that biological self-healing concrete can be effectively applied in regions with cold climates, maintaining the viability of bacterial spores within the concrete material.

### 3.2. Effect of the Curing Conditions

The survivability of bacteria in early-age concrete may be affected by the setting, hardening, and carbonation processes [18]. Water plays an essential role in early-age BSHC as it is required to harden concrete and revive bacterial spores. Water brings the dissolved mineral precursors and germinants necessary to transform inactive spores into active vegetative cells [34]. Due to the rapid change in concrete mechanical properties, pore size, pH levels, and amount of water, the number of viable spores in concrete may significantly decrease during the initial several days after BSHC specimen preparation [20]. Consequently, concrete curing conditions may be crucial for bacterial survivability in early-age concrete.

**Figure 6:**
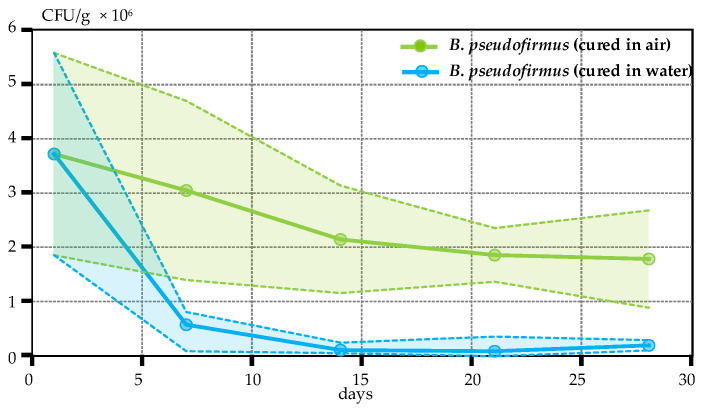
Bacterial viability in early-age concrete. Solid lines depict the mean values of the determined CFU/g, while the shaded regions indicate the 95% confidence intervals.

In the current research, we explored how the two prevalent curing environments, air and water curing, affect bacterial viability in early-age concrete. The calculated number of CFU in water and air-kept specimens is compared in Figure 6. As described in Section 2.5, specimens were separated into two groups starting from day 1: three specimens were kept in water and three in open-air conditions. After 7 days, the calculated number of CFU in water-kept specimens was almost five times lower in comparison to air-kept specimens. This difference reached more than ten-fold after two weeks and remained in this range up to the 28th curing day.

The obtained results clearly indicate that the bacterial survival rate in water-immersed specimens is much lower in comparison to the open-air conditions. Possibly, a large part of bacterial spores germinated into active cells several days after the concrete casting, as nutrients and germinants dissolved in water and became accessible for spores in water-kept specimens. However, in the absence of oxygen, the vegetative cells could not multiply, and most of them could die [35]. Contrarily, environmental conditions in the air-kept specimens were not suitable for early germination, and most of the initially EC-immobilized spores remained dormant. Tests reported in [36] did not show healing in the specimens incubated at RH 60% and 95%, indicating that such an amount of moisture was not sufficient to support the bacterial metabolism. Hence, water has a dual impact on the healing performance of BSHC elements. During the incubation period, it is essential for germination, metabolic activity of bacteria and precipitation of calcium carbonate into the crack [35]. At the same time, water may stimulate the early germination of bacteria in water-cured specimens. As concrete in the latter case remains uncracked, a large part of vegetative cells may die, reducing the self-healing ability of concrete.

### 3.3. Crack Healing

The experimental program for crack healing in BSHC elements was specifically designed to represent healing conditions that are similar to real structural applications. The reinforcing bars were placed at the bottom part of BSHC prisms, representing the structural reinforcement of beams. Thus, reinforcement carried the tensile stresses during the loading and allowed the formation of multiple cracks (Figure 2). The adopted experimental setup also allowed the creation of cracks of variable widths, similar to those observed in structural beams [37]. Moreover, specific rain-simulating healing conditions were employed for crack healing. These healing conditions were deemed to more accurately represent real-world scenarios compared to typical healing methods that use water immersion or environments with 100% relative humidity [35,36,38].

To evaluate crack closure over time, the healing ratio was selected as the main indicator of healing efficiency:

h = (w_i_ − w_t_)/w_i_ × 100%, (1)

where w_i_ is the initial crack width, and w_t_ is the crack post-healing width. Here, 100% healing refers to full closure of the initial crack.

**Figure 7:**
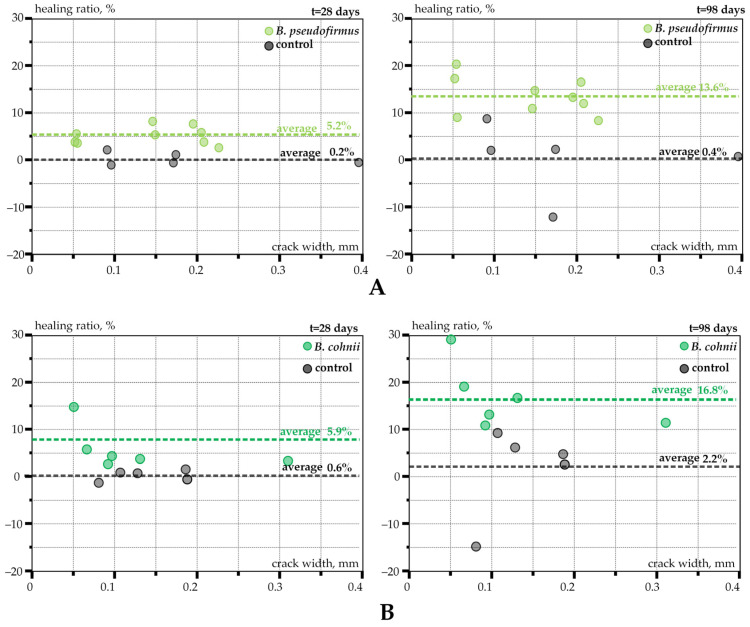
Variation in the healing ratio over time: (**A**) results of Test Series 1; (**B**) results of Test Series 2.

The healing ratio values for Test Series 1 and Series 2 samples are shown in Figure 7A,B, respectively. Here, each point on the graph represents the average value of crack width (calculated as an average from 10 locations and 30 measurement points).

Control specimens in both test series exhibited negligible healing ability with final healing ratios between 0.4 and 2.2%. On the contrary, several times higher healing ratios were observed in bacteria-containing specimens. The final healing ratio was equal to 13.6 and 16.8% for BSHC prisms of Test Series 1 and Series 2, respectively. However, the healing capacity of both test series was significantly reduced compared to our previous study [13], with healing ratios reaching up to 80%. The probable cause of such notable differences is attributed to the varied healing conditions: healing through immersion in water was applied in [13], whereas a rain-simulating environment was used in the present study. The flowing water could wash out the newly formed bacterial precipitation and loose aggregate, increasing the measured crack width. A comparable result was reported in [39], where biological concrete interacted with soil that was fully saturated. As a result of the water pressure, a substantial quantity of white precipitate emerged from the crack. The finding suggested that water circulation might diminish the healing effectiveness of BSHC components.

The experimental data obtained in the current study demonstrate that the incubation method has a significant impact on the healing ratios of BSHC specimens. As such, the healing capacity of open-air structures might be less effective than that of healing under idealized water-immersed laboratory conditions. The possible wash-out effect of newly formed precipitates must be taken into account when using bacterial concrete for structural applications.

## 4. Conclusions

The biological self-healing technique is an efficient and eco-friendly approach for damage management in concrete structures. Crack healing in BSHC elements is a result of bacterial metabolic activity, resulting in the deposition of calcium carbonate on the crack surfaces. Consequently, the capacity of BSHC to heal is closely dependent on the environmental conditions and the ability of bacteria to survive within concrete. The current study explored the impact of cold temperatures, freeze–thaw cycles, and curing conditions on the viability of bacteria. The study has shown that concrete-embedded bacterial spores can endure low temperatures and freeze–thaw (FT) cycles, retaining around 50% viability, making BSCH elements suitable for use in cold climates. Moreover, the survivability of bacteria is significantly impacted by curing conditions. Water immersion during curing can trigger early germination, decreasing viable spore counts by nearly tenfold. Air-curing under room conditions (50% relative humidity and 20 °C) provides better bacterial survivability, likely because spores remain dormant. The investigation showed that the self-healing performance of BSHC is heavily influenced by incubation conditions. Healing under simulated rain conditions showed significantly lower healing efficiency compared to water-immersed scenarios, indicating that the performance of BSHC in open-air environments may be inferior to controlled, idealized laboratory conditions. Future studies should investigate the long-term performance of BSHCs in real-world environments, particularly in regions with fluctuating temperatures and variable weather conditions, to validate laboratory results and improve practical applications.

## Figures and Tables

**Table 1:** Composition of BSHC.

Material	kg/m^3^	Mass Percentage
Portland cement CEM I 52.5 R (Aalborg White^®^)	463	26
Sand (0/4 mm)	855	49
Coated expanded clay particles with bacteria	270	15
Water	168	10

## Data Availability

Data are available from the authors upon request.
